# Behavioral Evaluation of Strengthened Reinforced Concrete Beams with Ultra-Ductile Fiber-Reinforced Cementitious Composite Layers

**DOI:** 10.3390/ma16134695

**Published:** 2023-06-29

**Authors:** Mohammad Iqbal Khan, Yassir M. Abbas

**Affiliations:** Department of Civil Engineering, College of Engineering, King Saud University, P.O. Box 800, Riyadh 11421, Saudi Arabia; yabbas@ksu.edu.sa

**Keywords:** ultra-ductile fiber-reinforced concrete, reinforced concrete, beam, ductility, strengthening, four-point test

## Abstract

In the literature, there is little information available regarding the behavior of composite beams made up of reinforced concrete (RC) and ultra-ductile fiber-reinforced concrete (UDFRC). In this study, UDFRC was examined for its effectiveness in enhancing the strength of RC beams. With a tensile strength of 4.35 MPa and a strain capacity of 2.5%, PVA-based UDFRC was prepared. The performance of 12 medium-sized reinforced concrete (RC) beams was measured under four-point flexural loading. The beams measured 1800 mm long, 150 mm wide, and 200–260 mm deep. The experimental program on beam specimens was divided into two phases. In the first, four 150 × 200 × 1800 mm RC beams with UDFRC layer thicknesses of 0, 30, 60, and 90 mm were tested. Additionally, four concrete and four concrete–UDFRC beams were investigated, measuring 150 × 230 × 1800 mm and 150 × 260 × 1800 mm, respectively. The study focused on medium-sized, slender RC beams under quasi-static loads and room temperature with additional or substituted UDFRC layers. As a result of replacing concrete with UDFRC, the load-carrying capacity at first crack and steel yield significantly increased between 18.4 and 43.1%, but the ultimate load-carrying capacity increased only in the range of 6.3–10.8%. Furthermore, beams with additional UDFRC layers could carry 30–50% more load than their concrete counterparts. An RC-UDFRC beam had a load-carrying capacity 10–15% greater than that of a comparable RC beam. Generally, there is a lower deflection response in UDFRC–concrete composite RC beams than in control concrete beams. The UDFRC layering can potentially improve the load-carrying capacity of RC beams, at least when ductility provisions are considered.

## 1. Introduction

### 1.1. Background

The construction industry overwhelmingly relies on RC structures due to their constructability and economic value. An estimated 25 billion tons of concrete are produced worldwide yearly (i.e., 3.8 tons of concrete made per person) [1]. However, its load-carrying capacity and durability are severely impeded by its brittleness under shear and tension. RC usually incorporates steel bars into the tensile regions to overcome this disadvantage. The tensile region of concrete is susceptible to cracking despite bar reinforcement, which compromises its durability. By incorporating discrete and random fibers into concrete to develop fiber-reinforced concrete (FRC), it becomes less brittle and less susceptible to damage from aggressive environments [2,3,4,5,6]. It is noteworthy that steel fibers are less susceptible to chloride corrosion than steel bar reinforcement; thus, they may be a better choice than steel bar reinforcement in chloride environments, and chloride salt attacks can accelerate the deterioration of concrete [7]. The success of conventional FRC has led to the development of a wide range of advanced fiber-reinforced cementitious materials. In recent years, the use of FRC has increased in mainstream construction, despite its decades-old history of introduction [8]. The use of concrete in structural applications exceeds 50% in developed countries [9]. These applications have led to a growing focus on the use of FRC in place of traditional reinforcement. It is generally recognized that FRC materials can either be strain-hardening or strain-softening, depending on their response to tensile stresses, typically based on the number and type of fibers used [10,11]. After the first crack of the material has occurred, strain hardening can be distinguished by an increase in tensile stress, and it is also accompanied by multiple cracks [12]. The University of Michigan researchers invented methods for adding about 2% fibers by volume to a normal strength matrix in order to obtain a strain-hardening composite [13,14,15].

The concrete industry has recently witnessed the emergence of ultra-ductile FRC (UDFRC), which has remarkable strain-hardening properties under tension [16]. The composite system was developed by integrating a cementitious matrix and polymeric fibers with a micro-mechanics design approach [14,17,18]. Due to its low fiber content and type, it has economic potential for practical applications. By controlling crack widths in the tensile region, UDFRC is predicted to improve composite beam durability and load-carrying capacity. The unique characteristics of UDFRC can lead to various structural applications that need to be explored. For an example of its potential performance enhancement applications, UDFRC could partially replace concrete in tension zones of structural members. With proper compatibility, the load-carrying capacity of beams could be improved as a result of the flexural resistance of the retrofitted layer [19,20,21]. In addition, this method could also be applied in situations where a large amount of bar reinforcement is required. As a result of its high tensile strength, UDFRC can improve the cost-effectiveness and constructability of a structure by reducing the problem of rebar congestion. The flexural resistance of the retrofitted layer would improve the load-carrying capacity of beams. Moreover, UDFRC serves as a better protective layer than regular concrete, enhancing the durability of the structural system [22]. The reason for this function is that the UDFRC can generally display multiple cracking behaviors under tension, with very small crack widths [19,23,24]. It is important to note that, in the literature, several terms have been used for UDFRC, including strain-hardening cement-based composites (SHCC), ductile fiber-reinforced cementitious composites (DFRCC), and engineered cementitious composites (ECC).

### 1.2. Overview of the Previously Published Works

Table 1 summarizes the most important studies on modifying RC- and concrete-beams with UDFRC layers. Several researchers have studied the application of UDFRC to reinforced concrete beams, and some studies investigated RC-UDFRC layered beams with different UDFRC layers for their flexural strength. The performance of RC-UDFRC layered beams with layer thicknesses between half and full beam height has been relatively understudied. Most UDFRC studies focus on layer thicknesses of more than 30% of the section height. There is also still uncertainty about how UDFRC thickness and reinforcement affect the flexural performance of the strengthened member. Further, many studies have been conducted on UDFRC structural members to investigate their flexural behavior, but relatively few studies have been conducted on RC beams strengthened with UDFRC. There is a strong possibility that these beams will be more economical and easier to use than conventional strengthening systems (e.g., by using RC and steel jackets, resins, etc.).

In the literature, there have been several studies that have been conducted on the efficiency of UDFRC as a retrofitting method for various structural elements. Few studies have focused on slabs, while most have addressed beams. Furthermore, very few studies have investigated ultra-high-performance Strain-Hardening Cementitious Composites (UHP-SHCC) as a strengthening technique for structural members. The investigation of SHCC-reinforced concrete beams was carried out by Khan and Abbass [1]. According to tests conducted by the authors, layering SHCC over the tension zone of the beams increases their load-carrying capacity. Similar results were reported by Zhang et al. [2], who showed that increasing the ECC layer thickness increased the flexural strength and ductility. Additionally, their numerical simulations show that ECC thickness exceeding a certain critical value will improve flexural strength and ductility significantly. A study by Yang et al. [3] investigated an SHCC-layered reinforced concrete beam with extensive polyethylene fiber-reinforced layers, as well as the cracking behavior with different layer thicknesses (20 and 40 mm). According to their experimental results, SHCC-layered RC beams have better flexural properties, crack width distributions, and crack spacing than conventional RC beams. In their numerical study [4], Shin et al. examined the effects of DFRCC strengthening in the tension region of RC beams. According to their findings, the strain capacity and strain-hardening slope of DFRCC are not significantly correlated with the load-carrying capacity and displacement at failure. A further study was conducted by Hussein et al. [5] to test the effectiveness of SHCC layers in strengthening RC beams over their soffit. By combining SHCC with small amounts of steel reinforcement, these scholars found that early strain localization was eliminated in the strengthening layer of the SHCC.

Furthermore, Anwar et al. [6] examined 21 plain concrete beams that contained predetermined artificial cracks and were repaired using carbon fiber-reinforced polymers (CFRP). A thin layer of ECC was found to be effective in replacing the inferior layer at the bottom of deteriorating beams, restoring the beams to their original condition. The failure of ECC substrates with CFRP pasted directly on them was also demonstrated to be the result of shear, rather than debonding at the interface. As well, Kim et al. [7] investigated the flexural performance of reinforced concrete beams reinforced with an SHCC and a high-strength reinforcing steel bar (HSRS bar), a new method for strengthening beams, taking into account both experimental data and numerical analysis. According to these authors, it was possible to control the crack width and improve the load-bearing capacity of beams by applying SHCC and HSRS bars. Additionally, Kamal et al. [8] applied ultra-high-performance strain-hardening cementitious composites (UHP-SHCC) to RC beams as a tensile-strengthening material. Under flexural loading, UHP-SHCCs achieved significant load-carrying capacity improvements. In a similar study, Basha et al. [9] compared several UHP-SHCC schemes for retrofitting cracked RC beams under flexure. As a result of their tests, UHP-SHCC proved to be an effective technique for increasing RC beams’ flexural strength and ductility.

The experimental investigation of twelve RC-ECC concrete beams reinforced with conventional reinforcement bars has been published in a study carried out by Shanour et al. [10]. In their study, the reinforcement percentages, fiber type (PVA and PP), and dosages were key variables. The results of their experiments showed that using PVA rather than PP with limited layer thickness significantly enhances the maximum load capacity. Moreover, Asgari et al. [11] describe experimental and numerical (section enlargement) methods for strengthening concrete beams with glass fiber-reinforced concrete. Experimental and numerical results demonstrated that using a glass fiber-reinforced concrete layer improves the load-carrying capability and ultimate deflection of concrete beams.

The effect of thin patches of high-strength SHCC on conventional RC beams has also been investigated by Wei et al. [12]. The test results show that strengthened RC beams have an increased ultimate shear capacity in comparison with reference beams. According to Nguyen et al. [13], composite beams made from SHCC and conventional concrete were also developed. As a result of their analysis, the tensile property was ranked highest in terms of its sensitive coefficient, followed by splitting tensile properties and, finally, compressive properties. A study by Khan et al. [14] has examined the effects of SHCC layers in tensile regions of flexural members under three different curing conditions (normal or hot curing or steam curing) in order to determine the enhancement of strength and flexural performance. This study demonstrated that normal curing regimes are effective and cost-effective and satisfy practicality, performance, and practicality requirements. In the context of other structural members, Chang-Geun et al. [15] have presented a series of empirical and numerical studies of SHCC (with 150–400 mm thicknesses) and RC slab systems. According to the study, the developed slab systems were more serviceable and capable of ductile than conventional RC slabs. More recently, Zhang et al. [16] and Zhang [17] have further investigated the behavior of RC members with flexural strengthening using SHCC. According to their findings, the thickness of the SHCC layer significantly influences the cracking behavior.

### 1.3. Importance, Objectives, and Scope of the Study

The available literature provides limited details about the behavior of RC-UDFRC composite beams. Therefore, the investigation of the effectiveness of UDFRC in strengthening and enhancing the structural performance of RC beams is presented. The research focused on how the UDFRC overlay thickness and percentage of bar-reinforcement affected the flexural and cracking responses of retrofitted beams. The flexural performance of composite beams was evaluated by measuring their deflection and strain under quasi-static loading. Furthermore, cracking analysis was performed on the beams to determine the impact of the UDFRC overlay.

**Table 1:** Summary of the literature survey.

Ref.	Type of Sample	Size of Sample (mm)			Retrofitting Layer			Insightful Conclusions
		L	D	B	Type	Thickness (mm)	Position	
[25]	RC beam	1200	250	150	UDFRC	25	Around the steel bars	o With the UDFRC layer, the load-carrying capacity slightly improved. o There was no significant difference in deflection behavior between the modified and control beams.
[26]	RC slab	2000	180	600	UDFRC	20 & 40	Slab’s soffit	o A substantial improvement in flexural strength has been achieved with UDFRC layering. o Compared with the control RC slab, the RC-UDFRC one has greater post-cracking stiffness.
[27]	RC beam	1200	160	160	UDFRC	25 & 50	Beam’s soffit	o Compared to the control RC beam, the RC-UDFRC beams had an increased maximum load-carrying capacity of 10% and a 34% deflection at failure. o The maximum stress of rebars decreases by 25–48% as the layer thickness increases. o Layered beams showed a 75–95% decrease in maximum crack widths when compared to control beams.
[28]	Concrete beam	500	100	100	Concrete	75 & 50	Beam’s soffit	o With an increasing UDFRC layer thickness, RC-UDFRC layered beams demonstrated a higher flexural strength.
					UDFRC	25 & 50		
[29]	RC beam	1480	170	130	UDFRC	20 & 40	Beam’s soffit	o The RC-UDFRC layered beams had several advantages over concrete beams in terms of flexural strength, crack width distribution, and flexural properties.
[30]	Concrete beam	400	100	100	UDFRC	10, 30m & 50	Beam’s soffit (around the crack)	o To increase the beam’s flexural strength, UDFRC can be substituted for concrete at the bottom by removing the weak layer.
[31]	Beam with high-strength reinforcing steel	3400	500	300	UDFRC	60	Beam’s soffit	o Compared with the control beam, the UDFRC layer provided slightly higher flexural strength. o Beams strengthened with UDFRC had better crack control than control beams and were less prone to deflection at peak loads.
[32]	RC beam	1800	200	200	Ultrahigh-performance UDFRC	50	Beam’s soffit	o Without rebar reinforcement, UDFRC overlays caused early strain localization, leading to brittle failures.
[33]	RC beam	1800	200	150	FRC	70	Beam’s soffit	o A thicker ultrahigh-performance UDFRC overlay beam has a higher strength and load-carrying capacity. o The overlay beams were observed to have a large number of fine cracks throughout their length compared to concrete overlay beams.
					Ultrahigh-performance UDFRC	30, 30, & 50		
[34,35]	RC beam	1200	150	100	UDFRC	10, 30, & 50	Beam’s soffit	o It was observed that the cracking response differed by layer thickness, with the thinner layers showing multiple cracks around the main crack of the RC layer. In contrast, multiple cracks exited randomly in a beam with a thicker substrate. o The UDFRC substrate contributed to sustaining the load and containing major cracks in the beams after the steel yielded.
[36]	RC beam	1850	280	115	UDFRC	100, 200, & 280	Beam’s soffit to full depth	o The UDFRC multi-crack behavior and strain hardening of the composite beam contributed most to the improved load-bearing capacity. o PVA-based UDFRC with 100 mm or 200 mm layer thicknesses increased the maximum load capacity by 20–25%.
[37]	Concrete beam	800	40	200	UDFRC	20	Beam’s soffit	o By dressing the bottom surface of concrete beams with fiber-based UDFRC layering, the test results demonstrate that the moment capacity of the beams at the ultimate phase can be notably enhanced.
[38]	RC beam	2000	200	100	Ultrahigh-performance UDFRC	20	Beam’s soffit with a length of 1.5 and 2.0 m	o UDFRC layering with bar reinforcement improved the cracking moment (by 28%) and ductility. o The flexural strength of a beam can be improved by casting a UDFRC layer along the span of the beam rather than stretching it along parts of it.
[39]	RC beam	2100 or 1500	350	180	High-tensile-strength UDFRC	20 (10/each face)	Beam’s side surfaces	o For span-to-depth ratios of 1.5 and 2.5, UDFRC increased the shear capacity by 13.8% and 18.8%, respectively. o The UDFRC layers on the strengthened beams contain fine crack widths. The UDFRC patches prevented the spalling of concrete.
[40]	RC beam	300	150	150	UDFRC	50, 75, 100, and 150	Beam’s top and bottom surfaces	o There was a very strong bond between concrete and UDFRC, even without anchoring. o It may be feasible to increase the load-carrying capacity further by placing UDFRC at the bottom of the beam versus the top.
[41]	RC beam	1650	250	150	UDFRC	50	Beam’s soffit	o The flexural behavior of RC-UDFRC beams depends heavily on curing conditions for the retrofitting layer. o Standard moist curing meets all practical, economical, and performance requirements.

## 2. Experimental Program

**Figure 1:**
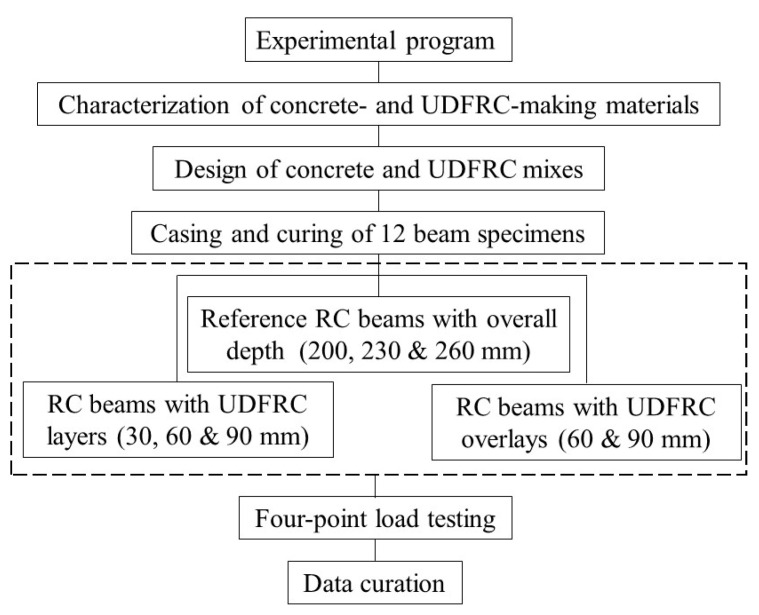
Variables and phases of the experimental program.

Two variables have been taken into account to achieve the objective of the investigation. The beam’s flexural behavior at different UDFRC layer thicknesses (e.g., 0%, 15%, 30%, and 45% of the beam’s overall depth) was studied. The thicker layer of UDFRC (i.e., 45% UDFRC layer depth) enables the minimization of the bar reinforcement. Two more sets of beams, one with reinforcements and one without, had UDFRC layer thicknesses of about 34% and 26% of the overall beam’s depth. These beams were also compared to their control counterparts. Figure 1 depicts a flowchart that outlines the study’s steps and variables.

### 2.1. Materials

#### 2.1.1. Cements and Chemical Admixture

**Figure 2:**
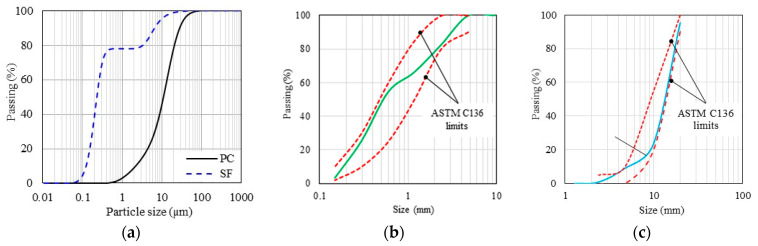
The GSD of: (**a**) cements, (**b**) blended sand, and (**c**) coarse aggregate. (Note. ASTM C136 [44]).

This study uses Portland cement type I (PC) as the main binder in concrete and UDFRC mixtures that satisfy ASTM-C150 [42] specifications. Moreover, fly ash (FA, class-F) was added to the UDFRC mixture as a supplementary cementitious material. Table 2 displays the cements’ physiochemical properties, while Figure 2 displays the distribution of their particle sizes. In this study, a modified polycarboxylic ether-based superplasticizer (SP) that meets ASTM C494 [43] type-F requirements was incorporated (about 1% wt. of PC) to achieve the desired workability (flowability of 180 ± 20 mm). There were approximately 0.36% solids in SP, while its relative density was 1.1.

**Table 2:** The physicochemical properties of the utilized cements.

Oxides (%)	PC	FA
SiO_2_	20.20	50.0
Al_2_O_3_	5.49	28.0
Fe_2_O_3_	4.12	10.4
CaO	65.43	<6
MgO	0.71	<4
SO_3_	2.61	2.5
Na_2_Oeq	0.26	1.5
Mean particle size (µm)	14	10
Loss on ignition (%)	1.38	4.0
Relative density	3.14	2.3
Fineness (m^2^/kg)	373	300–600

#### 2.1.2. Reinforcing Steel, Fibers, and Aggregates

The tensile reinforcement for beams was made from steel bars with a diameter of 16 mm in this investigation. Uniaxial tension tests according to ASTM A955 [45] were conducted to evaluate their mechanical properties. In the current investigation, UDFRC was developed using polyvinyl alcohol fibers (PVA). A Japanese company, Kuraray (Japan), produces these high-tensity fibers. In addition to their superior adhesion properties, these fibers are alkali-resistant and weatherproof. Table 3 provides the physiomechanical properties of PVA fibers.

In this study, cement-based mixtures were prepared using red-dune sand (RS) and white sand (WS), which are readily available in the Arabian Peninsula. The purpose of mixing RS and WS was to achieve adequate particle size gradation for concrete production that would result in the most favorable mechanical properties. Additionally, finely crushed silica sand (CS) was used as a fine aggregate to increase volume stability. As measured at saturated-surface-dry conditions, RS, WS, and CS had relative densities of 2.65, 2.74, and 2.70, respectively. The fineness modulus of RS and WS was 1.47 and 4.66, while the fineness modulus of their respective blends (65 and 35%, respectively) was 2.58. Concrete was made with coarse aggregates that could not exceed 10 mm in size. Figure 2 illustrates the grain size distribution of the employed aggregates, while Table 4 summarizes their physical characteristics.

#### 2.1.3. Composition Details of Mixtures

In this study, the concrete mixture was designed to achieve a 28 d compressive strength of 35 MPa. The UDFRC has been designed to exhibit multi-cracking behavior through a micromechanical approach. Further information on this method and the UDFRC mix design can be found in [46,47]. The quantities of the different constituent materials used for preparing concrete and UDFRC are displayed in Table 5.

### 2.2. Methodology

#### 2.2.1. Mixing, Casting, and Curing

This study involved pouring beams from the bottom to the top of the specimen mold with no UDFRC layers. It was, however, necessary to reverse the casting direction for RC beams containing layers of UDFRC. It is noteworthy that the casting direction has a significant impact on the old-to-new concrete interface bonding strength. In a study by Zhang et al. [48], it was found that the strongest bond occurred when the interface was located on top of the layering, followed by those located along the sides and at the bottom.

The casing of composite beams has been accomplished using the following method:

(a) Casting Group (A) beams (See Section 2.2.3):
  (i) A steel cage was first inverted, and concrete was placed in it to the desired height.
  (ii) A vibrator was used to remove any air bubbles from the concrete that had been poured.
  (iii) A wire brush was used to roughen the surface of the concrete after it had initially been set to ensure proper bonding between different layers of concrete.
  (iv) After the hardened concrete overlay was poured, a fresh layer of UDFRC overlay was applied to reach the target surface height. Several small layers of the mix were carefully placed for each UDFRC layer.
  (v) To achieve the desired reduction in air bubbles, the UDFRC layer was properly tapped.
(b) Casting Group (B) beams (See Section 2.2.3):
  (i) The steel cage was attached before the concrete or UDFRC was cast.
  (ii) A fresh layer of UDFRC overlay was poured to reach its target height. Several small layers of the UDFRC mix were carefully placed and tapped.
  (iii) A wire brush was used to roughen the UDFRC surface after it had initially been set to ensure proper bonding between different layers of concrete.
  (iv) After the hardened UDFRC overlay was poured, a fresh concrete overlay was applied and vibrated carefully.

A 28-day curing period was required for all beams under standard moist conditions after demolding.

#### 2.2.2. Testing of Materials

**Figure 3:**
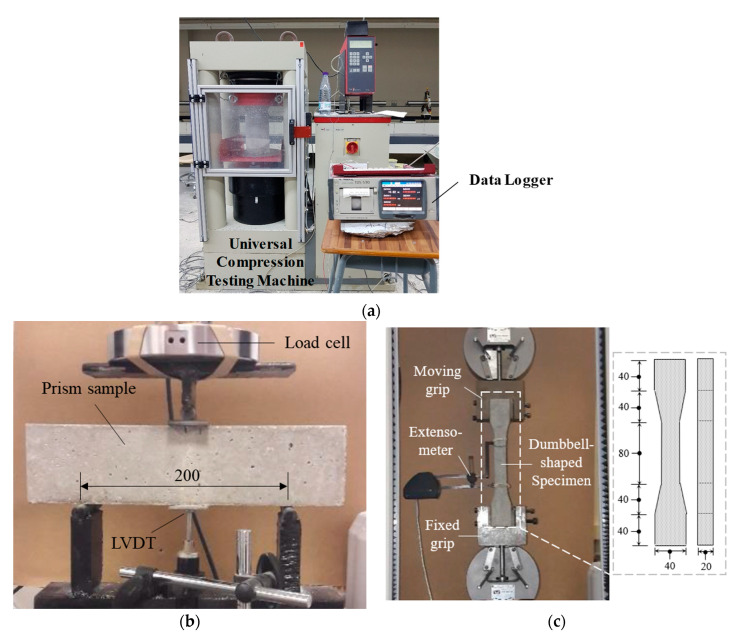
Testing details: (**a**) Uniaxial compression test, (**b**) three-point load test, (**c**) uniaxial tension test. (Dimensions are in mm.)

In this study, ASTM C39 [49] specifications were followed to measure the 28 d compressive strength with cylindrical specimens of 100 × 200 mm in size. This was accomplished by a ToniTechnic compressive strength testing machine (Figure 3a) with a 5.8 × 10^−3^ MPa/min loading rate. Under strain-controlled conditions (0.2 mm/min), the flexural strength was measured on 75 × 75 × 300 mm prisms (Figure 3b) using a universal testing machine (Instron–4637, 30 kN capacity). Moreover, the Instron–4637 machine was employed to measure the 28 d tensile behavior of UDFRC using dumbbell-shaped specimens (Figure 3c). The test was conducted under the displacement-controlled condition at a 0.15 mm/min rate.

#### 2.2.3. Details of Beam Specimens

**Figure 4:**
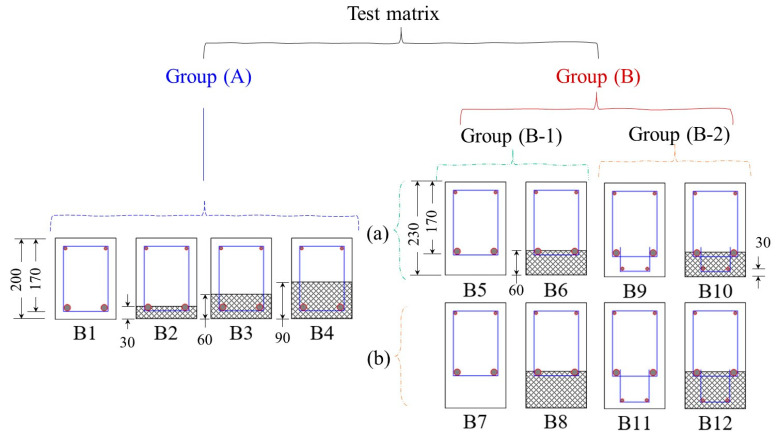
The shape of the beam specimens (See Table 6). (Note: All dimensions are in mm.)

In this study, the performance of 12 medium–sized RC beams [Figure 4] under four-point flexural loading was evaluated. These beams measured 1800 mm in length, 150 mm in width, and 200–260 mm in depth. It was necessary to keep the shear span–depth greater than two and design the beams with tension-controlled conditions and optimum transverse reinforcement to ensure pure flexural responses. The beams were thus reinforced longitudinally with two 16 mm diameter bars and two 8 mm hangers. Stirrups of 8 mm (with 90° standard hooks) were additionally placed at uniform intervals of 100 mm between the loading points. Over the course of the experimental campaign, the following phases were completed.

A. Casted and tested four 150 × 200 × 1800 mm RC beams (with thicknesses of 0, 30, 60, and 90 mm UDFRC). In this phase, the flexural behavior of the beams after replacing concrete with a UDFRC layer was studied.
B. The eight beams of this phase were divided into two groups:
  (1) Cast and test two 150 × 230 × 1800 mm RC beams containing 16 mm bars as the main reinforcement and two control beams (without UDFRC) as their counterparts.
  (2) Cast and test two 150 × 260 × 1800 mm RC beams containing 16 mm and 8 mm bars as the main reinforcement and two control beams (without UDFRC) as their counterparts.

**Table 6:** Details of the beam specimens.

Group	Set	Beam ID	h (mm)	C (mm)	U (mm)	$A_{s}$ (mm^2^)	ρ (%)	a/d
(A)	A	B1	200	200	-	402	1.34	3.06
		B2		170	30			
		B3		140	60			
		B4		110	90			
(B)	B-1-a	B5	230	230	-		1.17	
		B6		170	60			
	B-1-b	B7	260	260	-		1.03	
		B8		170	90			
	B-2-a	B9	230	230	-	502.5	1.46	2.90
		B10		170	60			
	B-2-b	B11	260	260	-		1.29	2.63
		B12		170	90			

$A_{s}$ = main reinforcement area; ρ=100 As/bh; C = Depth of concrete; U = Thickness of UDFRC substrate; a/d  = shear span–depth ratio.

#### 2.2.4. Testing of Flexural Behavior

**Figure 5:**
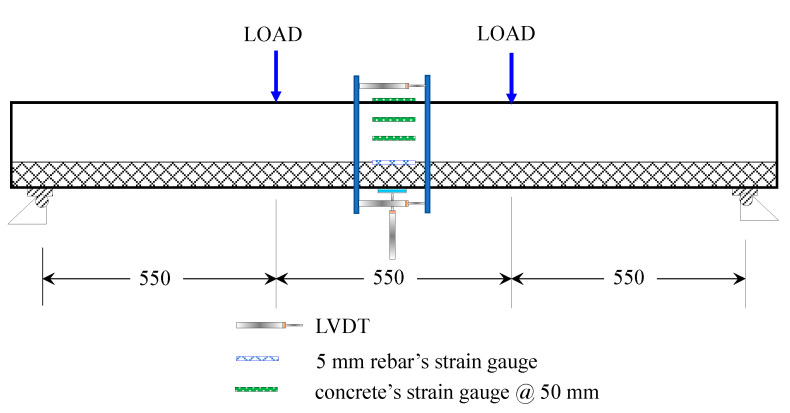
Four-point flexural test and instrumentation.

Figure 5 shows a detailed explanation of the setup for testing the RC beam under four-point loading. An AMSLER hydraulic testing machine with a 3000 kN capacity and a servo controller was used to subject samples to two monotonous loads distributed over 550 mm. Moreover, this figure illustrates the system of data acquisition accessories. The beam’s curvature was measured with two LVDTs mounted horizontally below and above the midspan, and its deflection was measured with an LVDT mounted vertically at its midspan.

Two strain gauges were fixed to its top surface to measure the maximum compressive strain of the beam. Furthermore, two strain gauges were positioned in the middle of the steel bars. Two strain gauges were also placed on the lateral face of the beam to monitor sectional strain variations. An X35 magnification crack detection microscope with a measuring range of 4 mm and a 0.02 mm division size was used to monitor cracks developing at multiple locations along the beam’s span.

## 3. Results and Discussion

### 3.1. Material Properties

In this study, uniaxial tension tests on three typical tension steel bars were conducted to determine their key mechanical properties. Based on the results of the tests (Table 7), it can be concluded that the steel used had an approximate yield strength of about 480 MPa, an approximate ultimate strength of about 623 MPa, and an approximate elasticity modulus of about 200 Gpa.

#### 3.1.1. Compression Test

The concrete specimens’ compression test showed an apparent initial elasticity modulus of 33Gpa and a mean characteristic strength of 40.3 MPa. The strains at the peak load and complete failure were 0.24 and 0.38%, respectively. Additionally, the mean characteristic compressive strength of the UDFRC was 57.8 MPa with strain at the peak stress of 0.21%. It is noteworthy that the beams in the present study were prepared in six batches. The compressive strength results obtained from these batches were fairly repeatable, with variable coefficients of about 6.4% and 6.7% for concrete and UDFRC, respectively.

#### 3.1.2. Uniaxial Three-Point Load and Uniaxial Tension Tests

**Figure 6:**
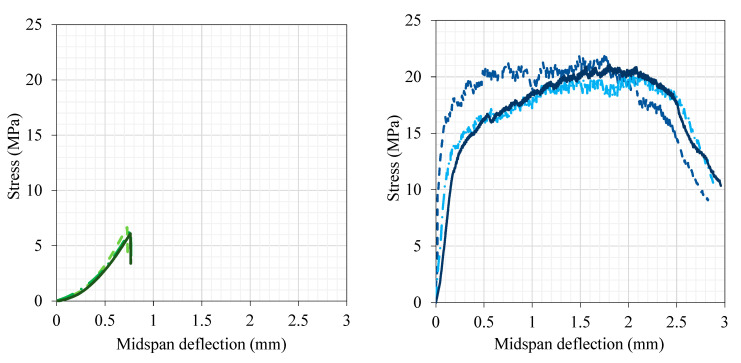
The behavior of concrete (**left**) and UDFRC (**right**) under three-point loading.

The response of three typical concrete and UDFRC prisms to three-point loading is illustrated in Figure 6. It can be seen from the figure that a strain-softening response with highly fragile failure occurs in concrete. In contrast, UDFRC showed strain-hardening and ductile behavior under flexural loading. According to these findings, the concrete’s modulus of rupture averaged 6.38 MPa, and the corresponding deflection was 0.74 mm. In comparison, the mean flexural strength and deflection at peak stress for the UDFRC were 20.5 MPa and 2.0 mm, respectively.

**Figure 7:**
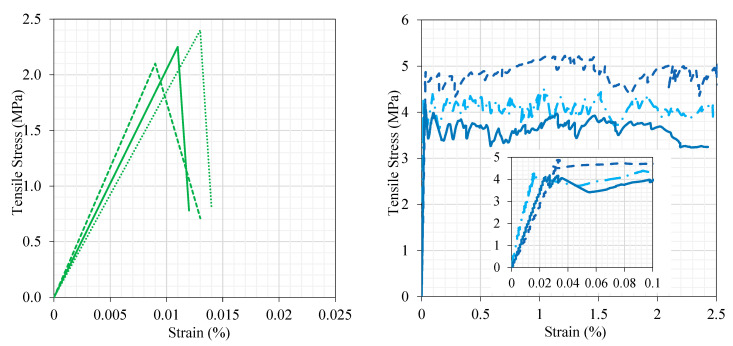
The behavior of concrete (**left**) and UDFRC (**right**) under uniaxial tension.

**Figure 8:**
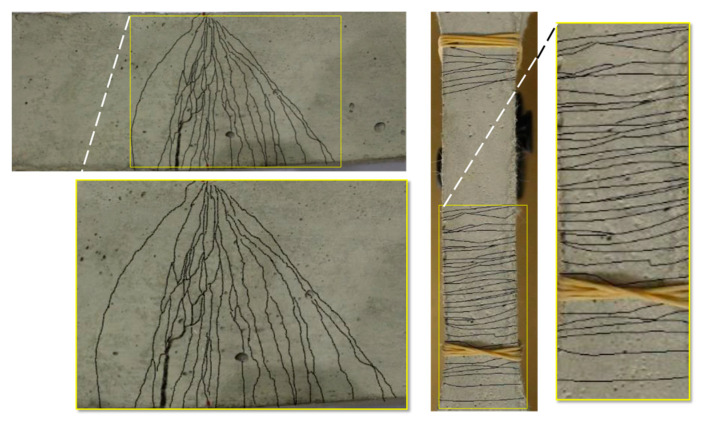
Multi-cracking response of UDFRC to the flexural (**left**) and tension (**right**) loading.

An illustration of the uniaxial tension responses of three typical concrete and UDFRC prisms is presented in Figure 7. A strain-softened and brittle behavior was observed in the concrete during flexural loading, whereas a strain-hardened and ductile behavior was observed in the UDFRC. The same behavior was seen under tensile loading as well. According to the results of the tests, UDFRC had an ultimate tensile strength of 4.35 MPa and a tensile strain capacity of 2.5%. According to Figure 8, UDFRC specimens exhibited a unique multi-cracking behavior (with micron-sized cracks smaller than 100 µm in size) under flexural and uniaxial tension loading. The tensile strength of the concrete was 2.25 MPa, while the corresponding strain was 0.011%.

### 3.2. Flexural Behavior of Beams

#### 3.2.1. Load-Deflection Behavior

**Figure 9:**
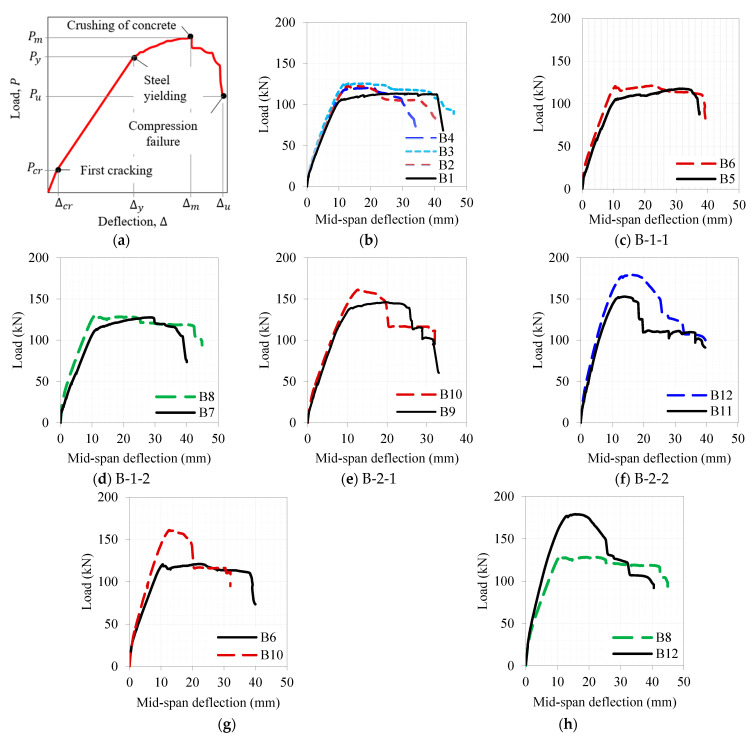
Load–deflection curves of the tested beams: (**a**) Typical response and that for sets (**b**) A, (**c**) B-1-1, (**d**) B-1-2, (**e**) B-2-1, (**f**) B-2-2, (**g**) B6 vs. B10, and (**h**) B8 vs. B12.

Figure 9a illustrates a typical load-deflection response for the tested beams (Table 6), a typical result for slender under-reinforced RC beams under flexural loading. The first characteristic of this response is its initial elastic behavior, which culminates in the initiation of a crack. The onset of this crack significantly decreases the stiffness of the beam up to the yielding of steel bars. The beam then shows nonlinear plastic behavior, which is accompanied by concrete crushing in most of the specimens examined. The analysis of the load-deflections parameters is summarized in Table 8. The values in parentheses indicate the percentage difference from the control beam.

The load–deflection curves of set-A beams (with substituted UDFRC layers of 0, 30, 60, and 90 mm thicknesses) are shown in Figure 9b. Based on the results of this study, the ultimate (failure) loads were approximately 80% of the maximum loads (i.e., $P_{u}=0.8P_{m}$ (see Figure 9a)). According to Figure 9b, set A beams generally behaved similarly. While the concrete–UDFRC beams’ cracked sections had a higher stiffness than the reference beam, the elasticity of the beams showed typical elastic characteristics (stiffness).

As can be seen in Table 8, replacing concrete with UDFRC notably increases $P_{cr}$ and $P_{y}$ (in the range 18.4–43.1%); however, little increase was caused in the load-carrying capacity (the increase was between 6.3 and 10.8%). This finding could be attributed to the high tensile strength of the UDFRC that enhances the load-carrying capacity in the elastic and cracked stages. However, the concrete–UDFRC bond failure may be responsive to the marginal increase in the load-carrying capacity at the ultimate stage. Further studies may address the impact of the surface treatment on the performance of concrete–UDFRC composite beams. It is noteworthy that this finding is consistent with some studies in the literature [31,50,51]. Likewise, this replacement seemed to delay cracking and steel yielding since the corresponding deflections were higher (19.2–56.6%) than those of the control beams. The concrete–UDFRC composite beams, however, showed premature maximum or ultimate loads. At these loads, deflections were notably smaller than those at control beam loads. This phenomenon is likely caused by the localization of the strain at the concrete–UDFRC interface [52,53].

The load–deflection curves in Figure 9c show the load–deflection curves for the 230 mm depth RC beam with a 60 mm UDFRC overlay (B6) and its reference case (e.g., no UDFRC, B5). B6 exhibited improved flexural performance compared to B5, especially in the elastic and cracking stages. Compared to the control beam, the pre-ultimate load-carrying capability of the beam was enhanced by 14.3 to 63.3% (see Table 8). Additionally, this table illustrates the positive effect of delaying steel yielding, thereby reducing deflection at a maximum load. As B6 approached its maximum load-carrying capacity, strain localization at the concrete–UDFRC interface resulted in delamination (see Section 3.2.1), resulting in an incredibly low resisting load. This may explain the convergence of the load–deflection curves at peak and ultimate loads. The discussion also applies to B7 and B8 (Figure 9d), where the beam’s overall depth was 260 mm, while B8 incorporated UDFRC of a 90 mm thickness.

It is evident from Figure 9e through Figure 9h that bar-reinforced UDFRC altered the beams’ flexural behavior, albeit with increased load-carrying capacity. These behaviors were evident in the deflection softening response and the lower deflection capacities of beams with UDFRC overlays than of those with plain UDFRC overlays. An over-reinforced (compression-controlled) beam will typically exhibit this load–deflection relationship. As a result, including a small amount of reinforcement within the concrete overlay could negatively affect the beam’s ductility. It may be possible to conduct in-depth research on the balance of reinforcement ratios in RC beams consisting of UDFRC layers in the future.

According to the UDFRC beams’ performance in load–deflection responses, their elastic stiffness and load-carrying capacities were significantly higher than those of the reference specimens (Figure 9). Additionally, the overlays with bar reinforcement could carry greater loads than those without them. In comparison with their comparable ones (B5, B7, B9, and B11, Table 8), the loads at the end-of-elasticity, yielding, and ultimate stages for B6, B8, B10, and B12 were approximately 44–83, 8–24, and 1–18% higher, respectively. Using a thicker overlay and additional rebar reinforcement would likely reduce the deflection capacity at different stages of loading. The deflections at end-of-elasticity were about 33–56% larger for A6, A8, A10, and A12 than for their analogous beams. A slight increase in deflection at yielding was observed for B8, B10, and B12 compared with the reference beams (2–4%), whereas the deflection at yielding decreased significantly for B6 (−17%). Furthermore, B6, B8, and B10 showed deflections at peak loads 24–41% lower than those of the reference beams, while B12 showed an increase of 14%. The deflection capacity of these beams was relatively close to their counterparts despite their lower deflections at maximum loads. The deflections measured when B6 and B10 failed were 10 and 23% less than those measured when B5 and B9 (the control beams) failed, whereas the values for B8 and B12 increased by 14 and 26% as compared to B7 and B11.

**Figure 10:**
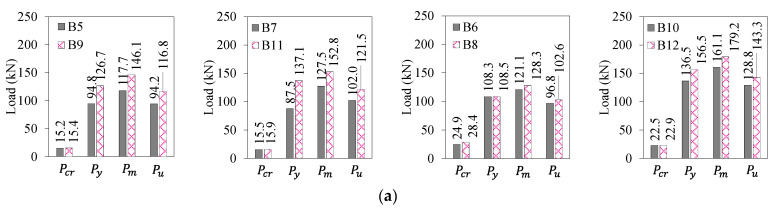

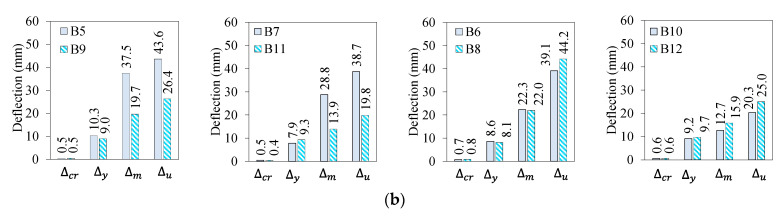
Load and deflection parameters for the concrete- and UDFRC-strengthened beams: (**a**) loads and (**b**) deflections.

This section also discusses the behavior of beams reinforced with concrete and UDFRC overlays. The critical deflection and load values for beams strengthened by 60 and 90 mm thick plain (B5 & B7) and reinforced concrete layers (B9 & B11) are presented in Table 8 and Figure 10. Compared with B5, B9 and B11 yielded higher yielding loads by 34–45% but smaller yielding loads by 8%. Similarly, the maximum loads of B7, B9, and B11 were 8–30% greater than those of B5. According to Table 8, the UDFRC-based strengthening in B10 and B12 led to 26 and 48% increased loads at the yield than in B6 and only about 10% more than in B8. Compared to B6, the maximum load-carrying capacities of B8, B10, and B12 were 7, 33, and 45% higher. As far as deflection at yielding is concerned, B8, B10, and B12 showed less deflection than B6, each by 24, 13, and 10%. A similar pattern was observed for B8, B10, and B12, where the peak load deflections were 23, 47, and 63% lower than those for B6. While B10 and B12 experienced higher deflections at steel yielding than B6, at 7% and 13%, respectively, they were less than that of B8 by around 6%. Furthermore, the maximum deflections for B8, B10, and B12 were lower than those for B6 by 1, 43, and 29%, respectively.

The above discussion showed that the UDFRC overlayed beams had a 30–50% higher load-carrying capacity than their concrete equivalents in the pre-ultimate loading phase. In comparison with normal RC layers (B9 and B11), UDFRC-bar-reinforced RC beams (B10 and B12) demonstrated a higher load-carrying capacity (by 10–15%). In contrast, the maximum loads of RC beams strengthened with plain UDFRC layers (B6 and B8) were marginally better (by about 2%) than those of their reference beams (B5 and B7). The deflection response of UDFRC–concrete composite RC beams was generally lower than that of concrete-only beams. This finding indicates that using UDFC layers would negatively impact the ductility of beams. A detailed analysis of the ductility of these beams will be discussed in Section 3.2.3. At least under conditions where the ductility provisions are considered, UDFRC layering will likely be an effective method of improving the load-carrying capacity of RC beams.

**Table 8:** The critical load–deflection parameters for the tested beams.

Set	Beam ID	Deflection (mm)				Load (kN)				Mode of Failure
		$\Delta_{cr}$	$\Delta_{y}$	$\Delta_{m}$	$\Delta_{u}$	$P_{cr}$	$P_{y}$	$P_{m}$	$P_{u}$	
A	B1	0.62	7.60	34.32	40.94	15.25	86.61	113.17	90.45	BY→CC
	B2	0.76 (+22.6%)	10.33 (+35.9%)	15.52 (−54.8%)	37.69 (−7.9%)	20.16 (+32.2%)	112.00 (+29.3%)	122.57 (+8.3)	97.12 (+7.4)	BY→DU→CC
	B3	0.82 (+32.3%)	9.52 (+25.3%)	17.27 (−49.7%)	42.75 (+4.4)	21.45 (+40.7%)	113.27 (+30.8%)	125.44 (+10.8)	98.81 (+9.2)	BY→DU→CC
	B4	0.97 (+56.6%)	9.06 (+19.2%)	20.28 (−40.9)	31.04 (−24.2)	21.82 (+43.1%)	102.54 (+18.4%)	120.30 (+6.3)	96.00 (+6.1)	BY→DU→CC
B-1-a	B5	0.49	10.31	37.45	43.62	15.23	94.78	117.72	94.18	BYCC
	B6	0.69 (+40.8%)	8.58 (−16.8%)	22.28 (−40.5%)	39.09 (−10.4%)	24.87 (+63.3%)	108.32 (+14.3%)	121.06 (+2.8%)	96.84 (+2.8%)	BY→DU→CC
B-1-b	B7	0.53	7.85	28.79	38.73	15.51	87.46	127.50	102.00	BY→CC
	B8	0.81 (+52.8%)	8.07 (+2.8%)	22.01 (−23.5%)	44.23 (+14.2%)	28.37 (+82.9%)	108.47 (+24.0%)	128.25 (+0.6%)	102.60 (+0.6%)	BY→DU→CC
B-2-a	B9	0.47	9.00	19.70	26.35	15.43	126.69	146.06	116.80	BY→CC
	B10	0.63 (+34.0%)	9.16 (+1.8%)	12.67 (−35.7%)	20.33 (−22.8%)	22.50 (+45.8%)	136.46 (+7.7%)	161.05 (+10.3%)	128.80 (+10.3%)	BY→DU→CC
B-2-b	B11	0.41	9.32	13.94	19.83	15.93	137.13	152.80	121.54	BY→CC
	B12	0.63 (+53.7%)	9.73 (+4.4%)	15.86 (+13.8%)	25.00 (+26.1%)	22.94 (+44.0%)	156.54 (+14.2%)	179.17 (+17.3%)	143.33 (+17.9%)	BY→DU→CC

#### 3.2.2. BY→CC: Bar Yielding and Concrete Crushing, and BY→DU→CC: Bar Yielding, Delamination of the UDFRC Layer, and Concrete Crushing Moment–Curvature Response

**Figure 11:**
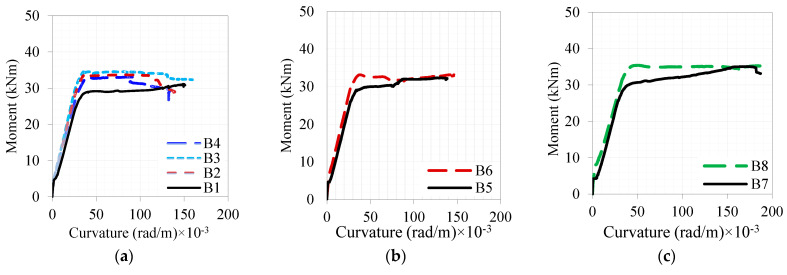

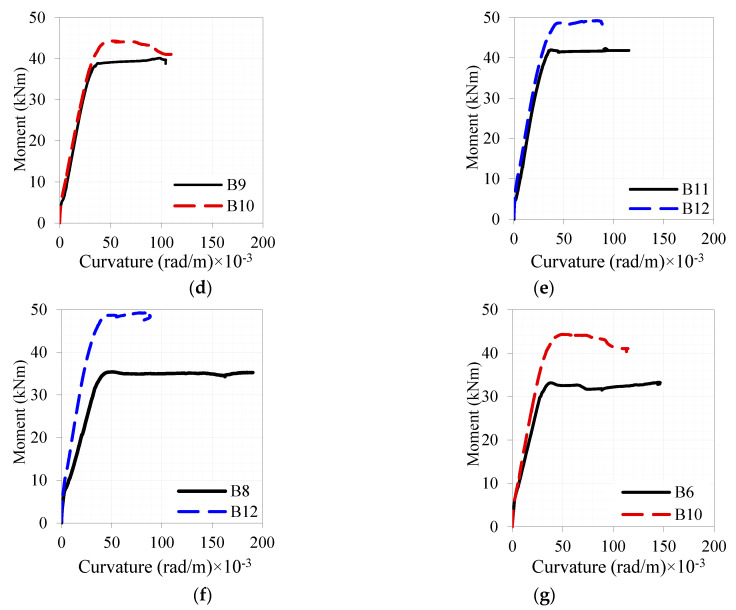
Moment–curvature curves of the tested beams: set: (**a**) A, (**b**) B-1-1, (**c**) B-1-2, (**d**) B-2-1, (**e**) B-2-2, (**f**) B8 vs. B12, and (**g**) B6 vs. B10.

Figure 11 displays the moment–curvature responses of the investigated beams to four-point loading. According to strain gauge measurements, the beam curvature after bending was calculated by assuming that the planar sections remain unchanged. The moment–curvature curves generally follow a similar pattern to the load–deflection curves for specimens. A summary of the key parameters of the moment (M)–curvature (ϕ) parameters is given in Table 9. Those parameters refer to the different types of typical flexural behavior described in Figure 9a. The beams with the UDFRC layers showed improved curvature deformability at both the elastic and cracked stages, as noted in the deflection responses. Despite this, these beams’ curvature responses are unlikely to exceed the curvature responses of reference beams at their ultimate or maximum loads. In a similar vein, it appears that the moment–curvature response follows the same logic as described for load–deflection.

The moment–curvature analysis (Figure 11 and Table 9) confirmed that UDFRC-strengthened beams showed significantly increased elastic stiffness and moment capacities compared to concrete-strengthened beams. Bar-reinforced layers on beams also increase their moment capacity as compared to plain ones, as expected. In general, however, the curvature capacity decreased as the layer thickness increased. The cracking, yielding, and maximum moments in B6, B8, B10, and B12 were 44–83%, 8–24%, and 1–16% higher than in control specimens (i.e., B5, B7, B9, and B11). Nevertheless, B6, B10, and B12 had higher cracking curvatures than their control specimens (B5, B9, and B11), while B8 had a lower crack curvature than its benchmark (B7). In B8, B10, and B12, the yielding curvatures were 8–17% larger than those of their counterparts, whereas, in B6, it was almost the same as in B5. A further observation was that the curvature of B6, B8, and B10 at the maximum moment was 15–50% less than that of comparable beams (i.e., B5, B7, and B9), and that of B12 was 46% greater than that of B11. In UDFRC-strengthened beams, the curvature at a peak moment was reduced compared to retrofitted concrete ones, yet the difference was insignificant at failure (around ±15%). Layered reinforced UDFRC beams generally exhibit a decreased curvature as their thickness increases. UDFRC reinforced with bars serves as a more effective strengthening material than regular concrete for the RC beams.

**Table 9:** The critical moment–curvature parameters for the tested beams.

Set	Beam ID	Curvature × 10^−3^ (rad/m)				Ductility	Moment (kNm)			
		$\phi_{cr}$	$\phi_{y}$	$\phi_{m}$	$\phi_{u}$	μ	$M_{cr}$	$M_{y}$	$M_{m}$	$M_{u}$
A	B1	1.60	25.58	149.90	149.90	5.86	4.20	23.82	31.09	31.09
	B2	2.20 (+37.5%)	29.46 (+15.2%)	82.04 (−45.3%)	139.50 (−6.9%)	4.74 (−19.1%)	5.50	30.80	33.70	29.00
	B3	2.70 (+68.8%)	27.97 (+9.3%)	81.97 (−45.3%)	159.66 (+6.5%)	5.71 (−2.6%)	5.90	31.15	34.54	32.31
	B4	3.00 (+87.5)	28.05 (+9.7%)	87.17 (−41.8%)	134.30 (−10.4%)	4.79 (−18.3%)	6.00	28.20	33.09	30.27
B-1-a	B5	2.00	27.4	145.0	152.3	5.56	4.19	26.1	32.4	31.9
	B6	2.84 (+42.0%)	27.2 (−0.7%)	123.3 (−15.0%)	123.3 (−19.0%)	4.53 (−18.5%)	6.84	29.8	33.2	33.2
B-1-b	B7	4.10	26.7	154.1	154.1	5.77	4.27	24.1	35.1	35.1
	B8	3.53 (−13.9%)	31.2 (+16.9%)	127.0 (−17.6%)	127.0 (−17.6%)	4.07 (−29.5%)	7.80	29.8	35.3	35.3
B-2-a	B9	1.28	28.3	98.0	104.3	3.69	4.24	34.8	40.2	39.4
	B10	2.10 (+64.1)	29.4 (+3.9)	49.1 (−49.9%)	114.5 (+9.8%)	3.89 (+5.4%)	6.18	37.5	44.3	41.0
B-2-b	B11	0.73	28.5	37.3	79.5	2.79	4.38	37.7	42.0	41.7
	B12	1.34 (+83.6)	30.7 (+7.7)	54.3 (+45.6)	88.1 (+10.8)	2.87 (+2.9)	6.30	43.1	48.6	48.6

#### 3.2.3. Ductility Analysis

In structural terms, ductility refers to the capacity of a member to sustain considerable inelastic deformations before it fails without significantly reducing its strength [54]. RC beams must have a certain level of ductility, a requirement outlined in several design codes (e.g., ACI 318 and EN-2). Typically, the curvature of a beam is determined by its curvature, deflection, or fracture energy response. This investigation used curvature ductility because it can be evaluated using only the sectional properties of the beam and is therefore more practical. Accordingly, we calculated the ductility parameter (μ) [55,56] by dividing the curvature at failure by the curvature at yielding (i.e., $\mu =\phi_{u}/\phi_{y}$) and summarized it in Table 9. This table indicates that B2–B4 (concrete–UDFRC composite beams) had inferior ductility compared to B1 (the control beam). This low degree of durability can be attributed to the delay in steel yielding in the composite beams with relatively no increase in curvature at failure (because of the failure of concrete–UDFRC bonding). Nonetheless, the beams exceeded the minimum strength threshold (3.32, as per [57]) for tension-controlled beams (with a reinforcement ratio no greater than 75% of the balanced one, as per ACI 318).

Table 9 compares the ductility parameters of UDFRC-strengthened beams with those of their control counterparts (B5–B12). The table shows that the ductility of the strengthened beams decreased with increasing UDFRC substrate thickness compared to the reference beams. The layer’s tension resistance may have reduced ductility (the section became compression-controlled due to over-tensile reinforcement in the presence of a considerable number of steel bars). However, most of the UDFRC-strengthened beams had adequate ductility parameters (i.e., above 3.32, as mentioned in [57]). Exceptions include concrete and UDFRC beams with 90 thicknesses (B11 and B12), with u equal to about 2.8, roughly −12% below the specified ductility threshold. In light of this finding and the improved load-carrying capacity (see Section 3.2.1), it can be concluded that the RC beam with a reinforced 60 mm bar substrate (B10) exhibited the most desirable flexural and ductility properties. This interpretation was made based on the fact that B10 was substantially more load-carrying and ductile than B9, and its ductility met the ACI 318 requirement for ample deformability warning before failure.

#### 3.2.4. Cracking Pattern and Failure Modes

**Figure 12:**
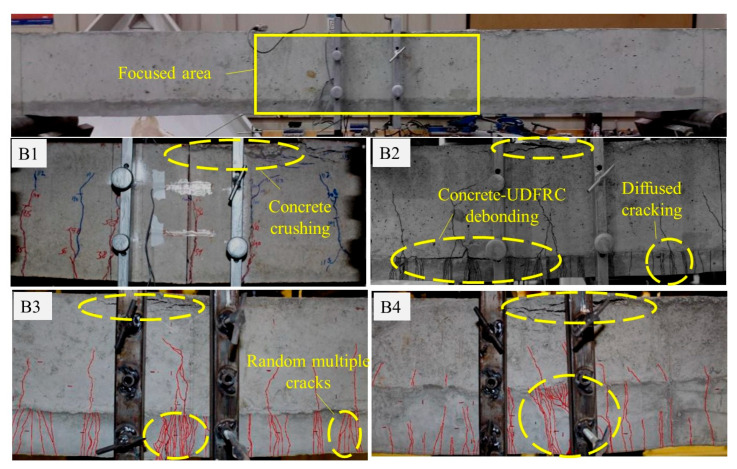
The cracking pattern of the beams of set A.

It is crucial for RC beams’ durability to control cracking responses and widths, especially in the bottom tension zones, where harmful substances can penetrate and compromise the beam’s structural integrity. This issue can be solved by UDFRC, because of its ultra-tiny multi-cracking properties. An illustration of the cracking response of the set-A beams is shown in Figure 12. The control beam (B1) displayed typical ductile cracking and failure patterns. This beam developed flexural cracks from its soffit along its top surface after the first crack appeared near the bottom of the midspan. A significant increase in crack width was observed following steel yielding. The beam failed when concrete crushing occurred on its top surface near its midspan (where compressive stress was maximal).

It was evident that crack widths were smaller in UDFRC layers than in concrete. Fibrous systems in UDFRC aid in stress bridging, which causes multiple cracks. As described in [58], multiple cracking can be classified as multiple random cracking or diffused multiple cracking. These cracking patterns were detected during yielding and ultimate loading (Figure 12). The UDFRC strain capacity was likely reached at maximum loading in B2, which led to the delamination of the concrete–UDFRC interface. The delamination occurred due to shear flow without effective bonding (i.e., no special surface treatment or shear reinforcement). Debonding between concrete and UDFRC was significantly shorter with thicker UDFRC layers. A thicker UDFRC layer also reduced the crack intensity significantly. This may be due to the significant depth of the UDFRC, which reduces strain localization and effectively controls crack development and propagation. As the control beam failed, all B2–B4 beams failed due to concrete crushing. The crack widths of the RC beams have been investigated further using a Java-based image processing program (ImageJ^®^, version 1.8.0) to study the effect of UDFRC on cracking. All beams containing UDFRC layers exhibited maximum crack widths of less than 100 µm at service loading (60–70% $P_{u}$). This is significantly smaller than the maximum specified by the ACI committee report 224R-01 [59] at 0.41 mm.

**Figure 13:**
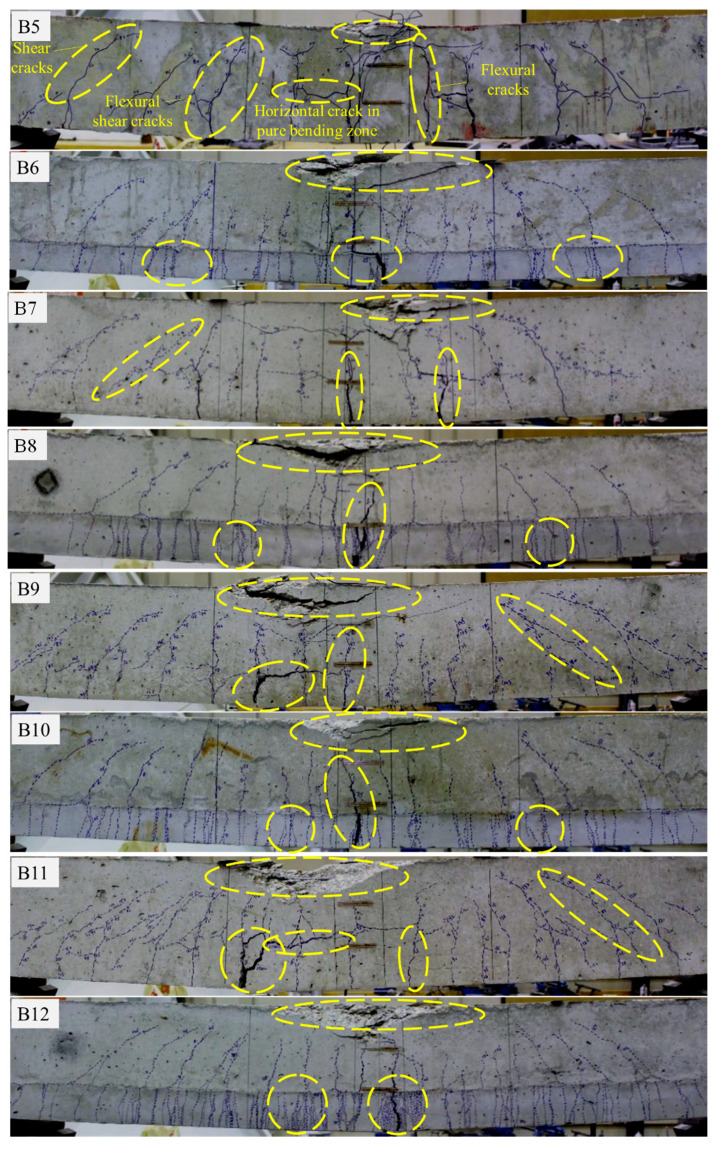
The cracking pattern of the beams of set B.

Figure 13 shows the cracking response of UDFRC-strengthened beams and their control counterparts (B5–B12) at the ultimate loading stage. In the concrete reinforced beams (B5, B7, B9, and B11), extensive flexural and shear-flexural cracks developed after the steel yielded. Noticeable horizontal cracks at the midspan of the beam and above the tensile reinforcement bars frequently accompanied the delamination of the concrete cover. A crack in UDFRC-reinforced beams (B6, B8, B10, and B12) typically originates at the surface of the concrete and propagates into the substrate. With the steel yielding, crack diffusibility over the substrate increased, and crack widths in concrete expanded vertically toward the beam’s top surface. Upon maximum loading and due to localized strains, the UDFRC substrate started to fracture. The compressive crushing of concrete near the midspan of the beam eventually caused the beam to collapse. Overall, a similar cracking pattern was observed in UDFRC substrates of B6, B8, B10, and B12 (i.e., random and diffuse multiple cracks). Additionally, the UDFRC-strengthened beams had narrower crack widths than the concrete-strengthened ones. There were no crack widths greater than 100 µm in UDFRC-strengthened beams under service loading conditions, which are significantly smaller than requirements for normal room conditions in ACI 224R-01 (410 µm). Another noteworthy observation was that cracks increased as UDFRC’s thickness and bar reinforcement increased.

## 4. Conclusions, Limitations, and Future Research

This study examined the impact of UDFRC substrate reinforcement and thickness on the flexural performance of RC-UDFRC composite beams. The composite beams were tested for their flexural response using four-point flexural loading. The focus of this study was the flexural behavior of tension-controlled medium-sized slender RC beams with added or replaced layers of UDFRC under quasi-static loading and normal room temperature. This research led to the following conclusions:

- The replacement of concrete with UDFRC of a 90 mm thickness notably increased the load-carrying capacity at first crack and steel yielding between 18.4 and 43.1%. However, no increase was observed in the ultimate load-carrying capacity (i.e., between 6.3 and 10.8%).
- Under pre-ultimate loading, UDFRC-overlayed beams could carry 30–50% more load than their concrete counterparts. The UDFRC-reinforced RC beams demonstrated a higher load-carrying capacity of 10–15% compared with normal RC beams. The maximum loads of reinforced RC beams with plain UDFRC layers were marginally higher than those of their reference beams.
- UDFRC–concrete composite RC beams generally displayed a lower deflection response than concrete-only beams. UDFRC layering can likely improve the load-carrying capacity of RC beams, at least when ductility provisions are taken into account.

In the future, researchers may conduct detailed studies of RC-UDFRC beams to explore the reinforcement ratio at balance conditions. By advancing RC-UDFRC beams through such research, the industry will be guided in the design of these beams, and therefore, their adoption will increase. Further, detailed studies may be conducted to determine the impact of surface treatment on the performance of RC-UDFRC composite beams.

## Figures and Tables

**Table 3:** Physicomechanical properties of the PVA fibers.

Length (mm)	Aspect Ratio	Density (g/cm^3^)	Tensile Strength (MPa)	Elasticity Modulus (gpa)	Elongation (%)
12	300	1.3	1600	42	7

**Table 4:** Physical properties of the used aggregates.

Physical Properties	RS/WS	CS	Coarse Aggregate
Unit weight (kg/m^3^)	1725	1552	1570
Relative density	2.63	2.68	2.65
Absorption (%)	0.77	1.52	1.45

**Table 5:** Composition of the concrete and UDFRC (kg/m^3^).

Material	Cement	FA	Water	Coarse Aggregate	RS	WS	CS	PVA Fibers
Concrete	350	-	175	1070	-	488	262	-
UDFRC	555	666	315	-	466	-	-	26

**Table 7:** Mechanical properties of the reinforcing steel.

Sample number	Strain (%)		Strength (Mpa)	
	Yielding	Rupture	Yield	Tensile
1	0.231	11.255	462.3	636.0
2	0.236	09.625	474.4	618.4
3	0.251	10.930	502.7	614.4
Mean	0.239	10.603	479.8	622.9

## Data Availability

Data is contained within the article.
